# Diagnostic value of circulating miRNA in the benign and malignant lung nodules: A systematic review and meta-analysis

**DOI:** 10.1097/MD.0000000000035857

**Published:** 2023-11-17

**Authors:** Li Liu, Fei Wang, Yan Nan, Xiaozhao Zou, Dan Jiang, Zhong Wang

**Affiliations:** a General Practice Department, Beijing Tsinghua Changgung Hospital, Changping District, Beijing, China.

**Keywords:** benign lung nodules, circulating miRNA, diagnostic accuracy, lung cancer, malignant lung nodules, meta-analysis

## Abstract

**Background:**

Lung cancer is the leading cause of death worldwide, and its diagnosis remains a significant challenge. Identifying effective methods to differentiate benign from malignant lung nodules is of paramount importance. This meta-analysis aimed to evaluate the clinical utility of circulating microRNAs (miRNAs) for the differential diagnosis of benign and malignant lung nodules.

**Methods:**

This study adhered to the Preferred Reporting Items for Systematic Reviews and Meta-Analyses guidelines. A comprehensive search was conducted across 4 electronic databases, without any temporal restrictions. The inclusion and exclusion criteria were strictly applied to assess the clinical applications of circulating miRNAs. A robust and transparent quality assessment was performed using the quality assessment of diagnostic accuracy studies-2 tool, and rigorous statistical analyses were conducted to synthesize the various diagnostic measures.

**Results:**

In the meta-analysis of 11 studies, quality assessment of diagnostic accuracy studies-2 assessment revealed < 5% high-risk methodologies, ensuring robustness. Sensitivity and Specificity were consolidated at 0.83 (95% confidence interval [CI]: 0.72–0.90) and 0.81 (95% CI: 0.73–0.88), respectively. The positive likelihood ratio and negative likelihood ratio were 4.45 (95% CI: 3.03–6.54) and 0.21 (95% CI: 0.12–0.35), respectively. The diagnostic odds ratio was 21.31 (95% CI: 10.25–44.30) and area under the receiver operating characteristic curve was 0.89 (95% CI: 0.86–0.91). Subgroup analysis highlighted significant variations in diagnostic accuracy by ethnicity and miRNA source, with non-Asian populations and serum-based tests showing higher diagnostic accuracy.

**Conclusion:**

This meta-analysis demonstrated that circulating miRNAs hold substantial diagnostic value in distinguishing between benign and malignant lung nodules.

## 1. Introduction

Lung cancer remains one of the most prevalent and lethal forms of cancer globally, and its stealthy nature poses significant challenges for early diagnosis and treatment. This issue is particularly acute in developing nations, where screening and diagnostic capabilities may not be as advanced as those in their developed counterparts. The worldwide disparity in the prognosis of lung cancer is telling. In China, the 5-year survival rate is only 16.10%, compared to 21.20% in the United States and 32.90% in Japan.^[1]^ This discrepancy illustrates not only the differences in healthcare infrastructure, but also the urgent need to implement early diagnostic strategies that are both effective and accessible. Low-dose computed tomography (LDCT) has emerged as a vital tool for lung cancer screening, particularly among high-risk populations. Although LDCT has increased the early detection rates, it is challenging.^[2]^ A staggering false-positive rate of 96.40% has been reported, leading to potential emotional distress and unnecessary medical expenditure.^[3]^ This reveals a critical need for improved diagnostic methods that can accurately differentiate between benign and malignant lung nodules.

Research has increasingly focused on the diagnostic potential of circulating microRNAs (miRNAs). These small, noncoding RNA molecules, which play pivotal roles in various cellular processes, have emerged as crucial components for understanding cancer biology.^[4,5]^ Their aberrant expression has been linked to cancer pathogenesis, reflecting the complexities of the genetic regulation and signaling pathways involved in tumorigenesis. The unique characteristics of miRNAs, such as their high stability in blood and sensitivity to changes in cellular microenvironments, make them promising biomarkers for lung cancer detection.^[4]^ The appeal of circulating miRNAs lies not only in their biological significance but also in their potential for noninvasive sampling, providing a window into the cellular changes that precede overt clinical manifestations. Several studies have reported differences in the plasma or serum miRNA expression profiles between patients with benign lung nodules and those with lung cancer. These findings have spurred interest in the possibility of using miRNA signatures to differentiate between malignant and nonmalignant lung conditions, thereby enhancing the diagnostic precision.

However, the road to clinical implementation has been fraught with inconsistencies in miRNA expression patterns, diagnostic thresholds, and experimental designs across studies.^[6,7]^ Variations in sample preparation, technology platforms, and statistical methodologies have contributed to conflicting findings. The complexity of miRNA biogenesis and function, coupled with interpatient variability, further complicates the interpretation of these results. These challenges highlight the need for a comprehensive analysis that not only synthesizes existing data, but also addresses the methodological heterogeneity that has hampered progress. This systematic review and meta-analysis aimed to consolidate existing research on the clinical applicability of circulating miRNA expression profiles in the diagnosis of benign and malignant lung nodules. By synthesizing evidence from diverse sources, we sought to provide an authoritative reference that could guide clinical practice and future research in this promising yet complex field.

## 2. Materials and methods

### 2.1. Search strategy

In the execution of this meta-analysis, the methodology was meticulously aligned with the Preferred Reporting Items for Systematic Reviews and Meta-Analyses (PRISMA) guidelines.^[8]^ A comprehensive search was conducted on January 6, 2023, across 4 electronic databases, including PubMed, Embase, Web of Science, and Cochrane Library, without any temporal restrictions. The search strategy was tailored to each database with specific vocabulary and syntax adapted accordingly. The following search strings were used for PubMed: (lung cancer OR lung adenocarcinoma) AND (diagnosis [Title/Abstract]) AND (miRNA OR miRNA OR pre-miRNA) AND (serum OR plasma OR circulatory). The search was unrestricted by language and ensured a broad and inclusive review. Furthermore, the reference lists of pertinent articles were manually scrutinized to identify any additional relevant records that may have contributed to the analysis.

### 2.2. Inclusion criteria

Studies included in the systematic review were required to meet the following criteria: Studies that evaluated the clinical utility of circulating miRNAs in the differential diagnosis of benign and malignant lung nodules; Studies that utilized histological examination as the gold standard for diagnosing benign or malignant lung nodules; Studies that provided sufficient data to calculate the number of true positives, false positives, false negatives, and true negatives; and Studies that included both benign and malignant lung nodule groups.

The exclusion criteria were as follows: Studies in which the control and study groups consisted solely of healthy individuals and lung cancer patients, without involving benign lung nodules; Documents with incomplete or unclear analytical data and inconsistent outcome indicators; and Reviews, basic experimental studies (including animal and cellular studies), abstracts, and conference summaries.

### 2.3. Data extraction

Literature screening and data extraction will be performed independently by 2 evaluators and cross-verification will be conducted. Should any discrepancies arise during this procedure, conflicting issues will be resolved through discussion between the involved reviewers, and a third evaluator may be consulted if necessary. The specific data to be extracted encompassed the following aspects: basic information of the study, such as the first author, country, and publication year; fundamental sample data, including mean age, total sample size, sex distribution, ethnicity, and the particular miRNA profile utilized for diagnosis; and sensitivity and specificity measures. In instances where the desired data are not found in the published report, contact will be made with the original study’s investigators via email to request unpublished information, ensuring a comprehensive and accurate meta-analysis.

### 2.4. Quality assessment

The methodological quality of the studies included in this meta-analysis was rigorously appraised using the quality assessment of diagnostic accuracy studies-2 tool.^[9]^ This instrument was specifically designed to assess the quality of diagnostic accuracy studies and comprised 4 primary domains: Patient Selection, Index Test, Reference Standard, and Flow and Timing. The risk of bias was methodically assessed in each of these domains, and concerns regarding applicability were considered in the first 3 domains. Signaling questions were used to probe each domain, and the responses were categorized as “yes” for a low risk of bias or concern, “no” for a high-risk of bias or concern, or “unclear” if the information was not clearly stipulated. This multifaceted approach offers a robust and transparent framework for quality assessment, thus ensuring the integrity and reliability of meta-analysis findings.

### 2.5. Statistical analyses

Statistical analyses for this meta-analysis were conducted using an established and rigorous methodology. Stata version 17 (StataCorp, College Station, TX) was employed to synthesize the sensitivity, specificity, positive likelihood ratio (PLR), negative likelihood ratio (NLR), and diagnostic odds ratio (DOR), along with their respective 95% confidence intervals (CIs). Heterogeneity among the studies was assessed using the chi-squared (χ^2^) test, and an *I*^2^ statistic > 50% was considered indicative of substantial heterogeneity. When heterogeneity was prominent, a random-effects model was used to combine the statistical measures, whereas a fixed-effects model was employed when heterogeneity was lower. In cases of significant heterogeneity, the extent of the threshold effect was evaluated using the Spearman rank correlation coefficient between the logit of sensitivity and logit of (1-specificity). Further investigations into the sources of heterogeneity were conducted using sensitivity analysis, subgroup analysis, and meta-regression. Publication bias was assessed by using funnel plots. The level of statistical significance was set at a 2-tailed α-value of 0.05.

## 3. Results

### 3.1. Search results and study selection

**Figure 1.**
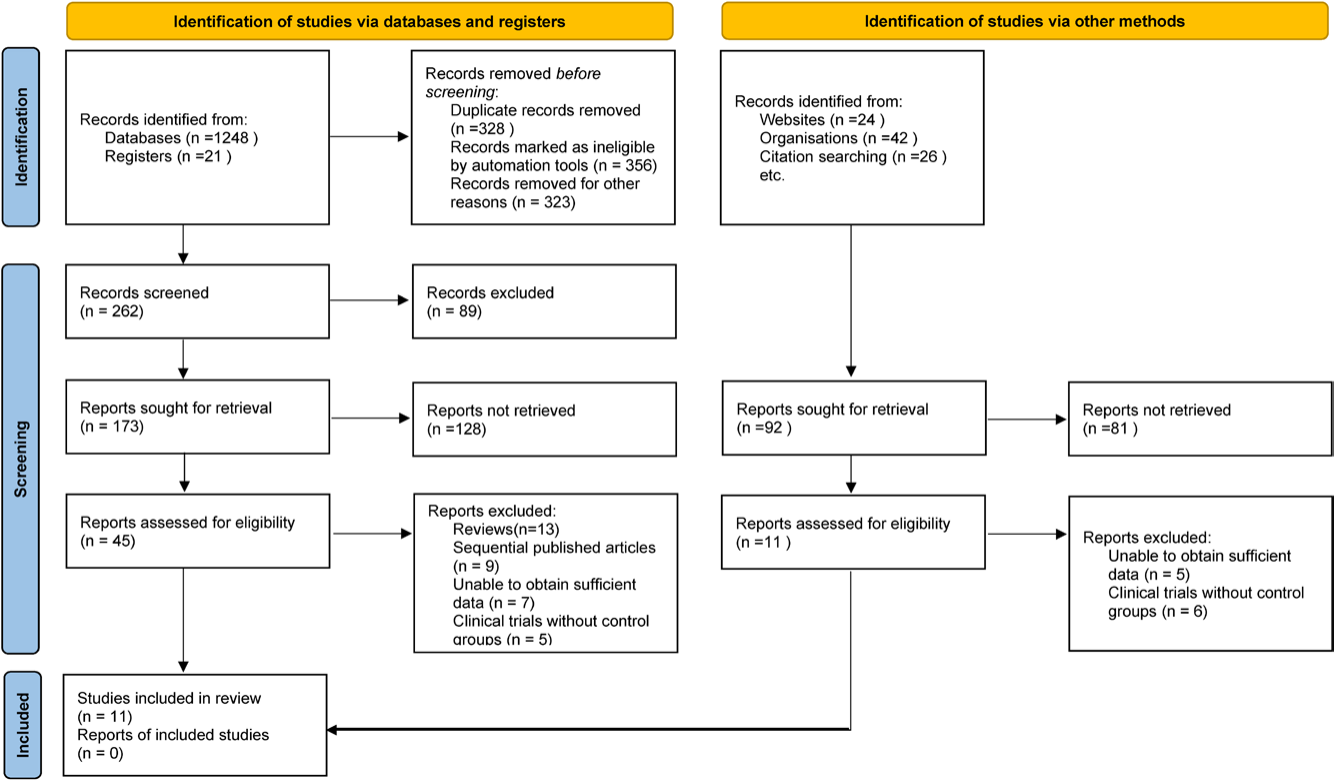
Selection process of included studies.

A total of 1269 pertinent studies were initially identified at the commencement of the electronic database search. Following the removal of duplicates and careful examination of titles and abstracts, 45 relevant studies were isolated. Strict adherence to the predefined inclusion and exclusion criteria led to the exclusion of 34 studies upon further scrutiny. Ultimately, 11 articles met all the criteria and were included in the analysis, as referenced in the citations.^[6,7,10–18]^ The entire literature screening process, along with the resulting outcomes, is depicted in Figure 1.

### 3.2. Study characteristics

This meta-analysis included 11 studies conducted between 2011 and 2019. The majority of the studies originated from China, representing both Asian, Caucasian, and African ethnicities, with additional contributions from Italy, Japan, and the USA. The sample sizes among the studies varied, with case and control numbers ranging from 20/19 to 274/122. Various miRNA expression patterns have been investigated, with some studies focusing on specific miRNAs that are either upregulated or downregulated, whereas others have explored a broader set of miRNAs. The sources of miRNAs analyzed in these studies were predominantly plasma and serum (Table 1).

**Table 1:** Characteristics of studies included in the meta-analysis.

Included study	Year	Country	Ethnicity	Sample size (case/control)	miRNA expression pattern	Source of miRNA
Cazzoli et al	2013	Italy	Caucasian/African	50/30	miR-151a-5p, miR-30a-3p, miR-200b-5p, miR-629, miR-100, miR-154-3p (Upregulated)	Plasma miRNA
Fan et al	2018	China	Asian	70/22	miR-15b-5p, miR-20a-5p, miR-19a-3p, miR-92a-3p, miR-16-5p (upregulated), miR-146b-3p (downregulated)	Serum miRNA
He et al	2018	China	Asian	274/122	hsa-miR-199a-3p, hsa-miR-148a-3p, hsa-miR-210-3p, hsa-miR-378d, hsa-miR-138-5p	Serum miRNA
Li et al	2017	China	Asian	20/19	miRNA-21-5p, miRNA-574-5p, CEA and CYFRA21-1 (Upregulated)	Serum miRNA
Lin et al	2017	China	Caucasian/African	63/63	miRs-126, 210 and 205-5p (upregulated)	Plasma miRNA
Shen et al	2011	USA	Caucasian/African	32/33	miR-21, miR-210 (upregulated) and miR-486-5p (downregulated)	Plasma miRNA
Tai et al	2016	Japan	Asian	110/47	a set of 20 miRNAs	Serum miRNA
Tang et al	2013	China	Asian	34/30	miR-21, miR-155 (upregulated) and miR-145 (downregulated)	Plasma miRNA
Wang et al	2015	China	Caucasian/African	108/56	miR-483-5p, miR-193a-3p, miR-25, miR-214 (upregulated)	Plasma miRNA
Xi et al	2018	China	Asian	42/15	miRNA-182 (upregulated)	Plasma miRNA
Zhang et al	2019	China	Asian	Aug-32	miR-185-5p, miR-32-5p, miR-140-3p and let-7f-5p	Plasma miRNA

miRNAs = microRNAs.

### 3.3. Results of quality assessment

**Figure 2.**
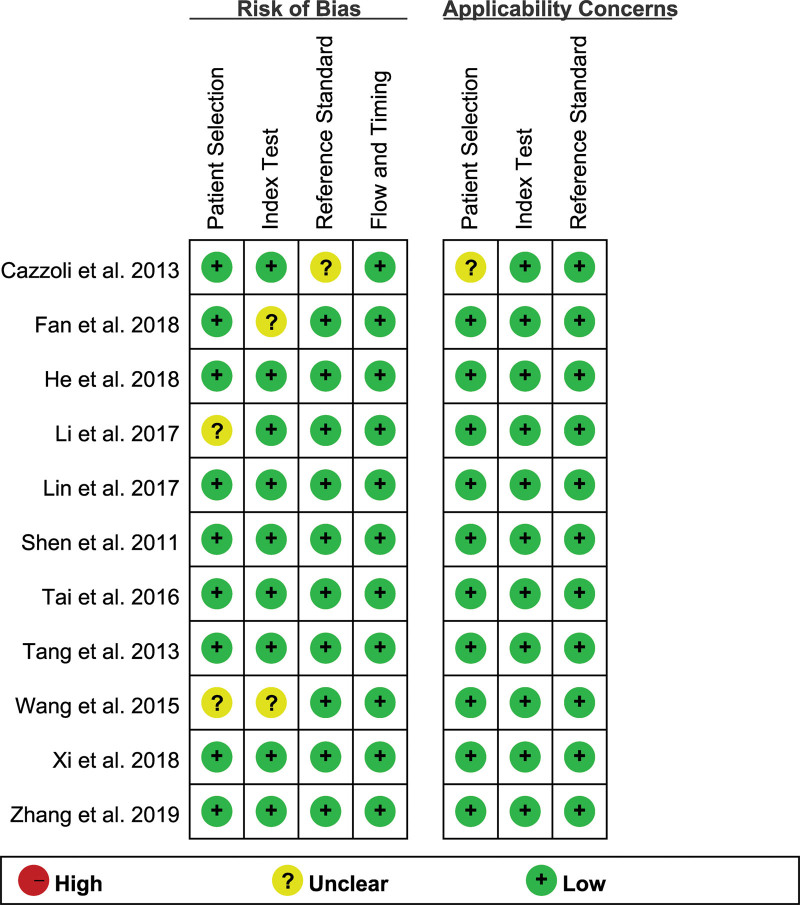
Risk of bias and applicability concerns summary.

The results of the quality assessment of diagnostic accuracy studies-2 assessments for each included study are shown in Figure 2, providing a detailed visualization of methodological quality across 4 essential domains: patient selection, index test, reference standard, and flow and timing. Impressively, the proportion of high-risk patients in each of these critical domains was found to be < 5%. Such a low percentage of high-risk patients signifies rigorous adherence to research methodologies, thereby enhancing the overall credibility of the included studies.

### 3.4. Meta-analysis results of sensitivity and specificity

**Figure 3.**
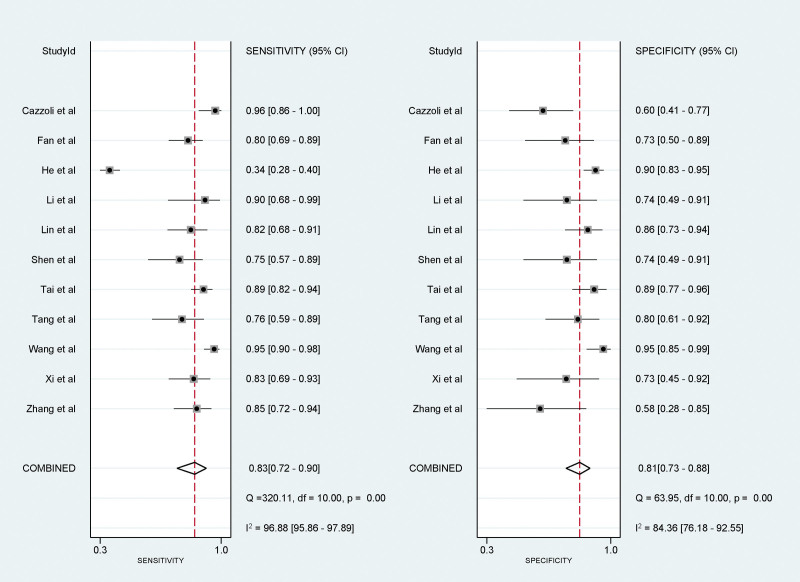
Forest plots depicting the sensitivity and specificity of circulating microRNA in differentiating benign from malignant pulmonary nodules.

We employed a random-effects model to ensure a more generalized inference of the findings. Sensitivity, an essential measure of how well the test identifies true positive cases, was amalgamated at 0.83 (95% CI: 0.72–0.90). This indicates that the test correctly diagnosed the condition in 83% of individuals who actually had it. Specificity, on the other hand, measures how well the test identifies true negative cases, and it was consolidated at 0.81 (95% CI: 0.73–0.88), signifying that 81% of the healthy individuals were correctly identified (Fig. 3).

### 3.5. Meta-analysis results of PLR and NLR with random-effects model

**Figure 4.**
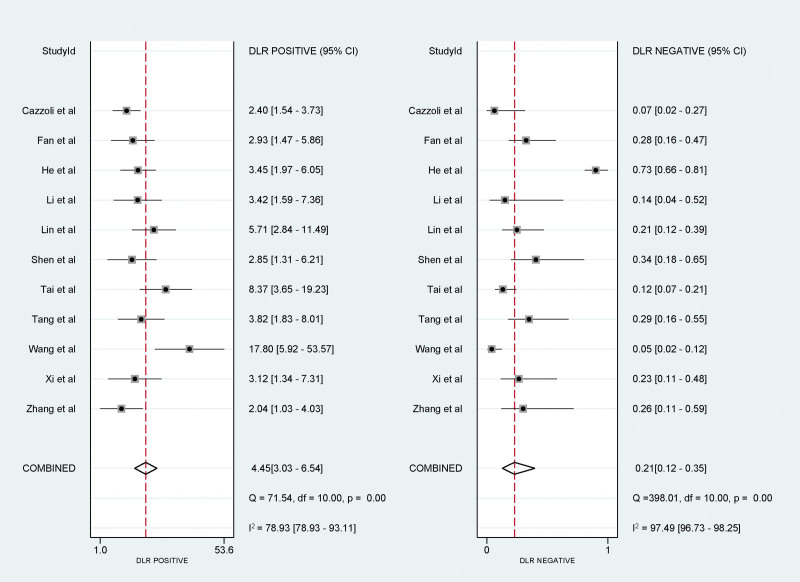
Forest plots depicting the positive and negative likelihood ratios of circulating microRNA in differentiating benign from malignant pulmonary nodules.

The PLR was merged at 4.45 (95% CI: 3.03–6.54), denoting the ratio of the probability of a positive test result in diseased versus non-diseased individuals. A higher PLR emphasizes the reliability of the test for confirming the condition. The NLR was consolidated at 0.21 (95% CI: 0.12–0.35), reflecting the probability ratio of negative test results between the diseased and non-diseased groups. A lower NLR strengthened the diagnostic ability of the test to exclude the disease (Fig. 4).

### 3.6. Meta-analysis results of DOR with random-effects model

**Figure 5.**
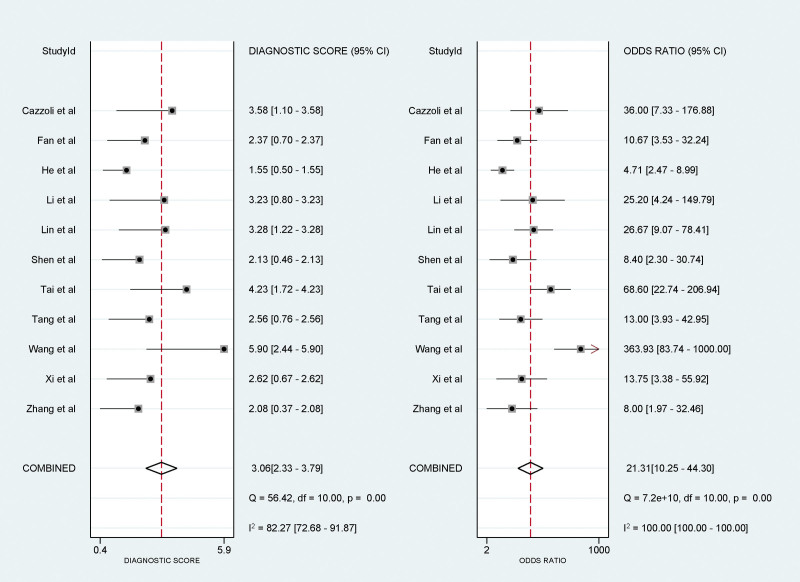
Forest plots depicting the diagnostic score and diagnostic odds ratio of circulating microRNA in differentiating benign from malignant pulmonary nodules.

DOR, a robust measure that encapsulates both sensitivity and specificity, was amalgamated at 21.31 (95% CI: 10.25–44.30). A higher DOR suggests that the diagnostic test is effective in differentiating between the diseased and non-diseased states (Fig. 5).

### 3.7. Meta-analysis results of area under curve (AUC)

**Figure 6.**
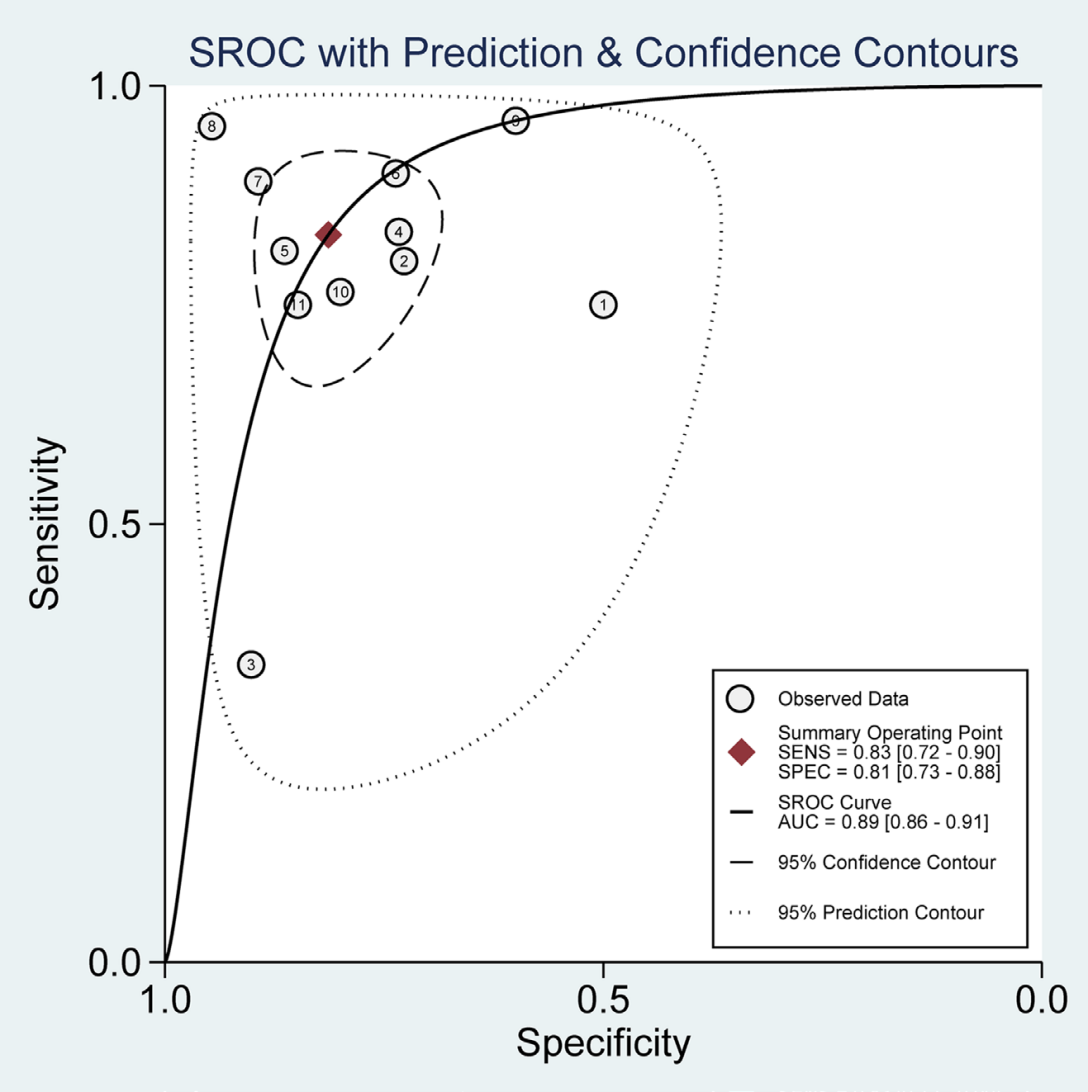
Summarized receiver operating characteristic curve of circulating microRNA in differentiating benign from malignant pulmonary nodules.

The AUC, an integrative evaluation of the test’s overall accuracy, was determined to be 0.89 (95% CI: 0.86–0.91). This value emphasizes the ability of the test to correctly classify individuals into diseased or non-diseased categories (Fig. 6).

### 3.8. Subgroup analysis of diagnostic indicators by ethnicity, miRNA spectrum, and miRNA source

The results of subgroup analysis indicated statistically significant variations in diagnostic accuracy in the differentiation of benign and malignant lung nodules across ethnicities, miRNA spectra, and miRNA sources. The diagnosis of lung nodule malignancy using miRNAs exhibits different accuracies among various ethnicities. Specifically, the AUC was higher in the non-Asian population (AUC = 0.92, 95% CI: 0.91–0.95) than in the Asian population (AUC = 0.85, 95% CI: 0.83–0.86). In the context of the miRNA spectrum, the diagnostic accuracy for lung nodule malignancy in the single miRNA category (AUC = 0.81, 95% CI: 0.78–0.82) was lower than that in the combined miRNA category (AUC = 0.88, 95% CI: 0.86–0.90). Regarding miRNA sources, the diagnostic accuracy for miRNAs in plasma (AUC = 0.85, 95% CI: 0.83–0.87) was lower than that for miRNAs in serum (AUC = 0.88, 95% CI: 0.85–0.93, Table 2).

**Table 2:** Subgroup analysis of diagnostic accuracy based on ethnicity, miRNA spectrum, and miRNA source.

Category	Sensitivity	Specificity	PLR	NLR	DOR	AUC
Ethnicity						
Asian	0.65 (0.60–0.67)	0.79 (0.75–0.81)	3.35 (2.55–4.20)	0.28 (0.18–0.40)	11.90 (7.20–16.70)	0.85 (0.83–0.86)
Non-Asian	0.87 (0.81–0.90)	0.82 (0.81–0.88)	5.20 (2.75–9.50)	0.18 (0.07–0.25)	36.00 (16.50–89.90)	0.92 (0.91–0.95)
miRNA spectrum						
Single	0.76 (0.73–0.82)	0.77 (0.70–0.85)	3.30 (2.05–5.80)	0.29 (0.17–0.35)	12.50 (6.00–30.00)	0.81 (0.78–0.82)
Combined	0.70 (0.68–0.73)	0.78 (0.76–0.81)	3.90 (2.90–5.30)	0.24 (0.12–0.33)	18.60 (12.00–34.50)	0.88 (0.86–0.90)
miRNA source						
Plasma	0.74 (0.70–0.77)	0.74 (0.70–0.78)	3.20 (2.40–4.40)	0.27 (0.18–0.35)	12.40 (8.00–21.70)	0.85 (0.83–0.87)
Serum	0.64 (0.61–0.68)	0.84 (0.80–0.88)	4.20 (2.50–6.70)	0.22 (0.06–0.50)	20.80 (7.20–68.50)	0.88 (0.85–0.93)

AUC = area under curve, DOR = diagnostic odds ratio, NLR = negative likelihood ratio, PLR = positive likelihood ratio, miRNAs = microRNAs.

### 3.9. Publication bias

**Figure 7.**
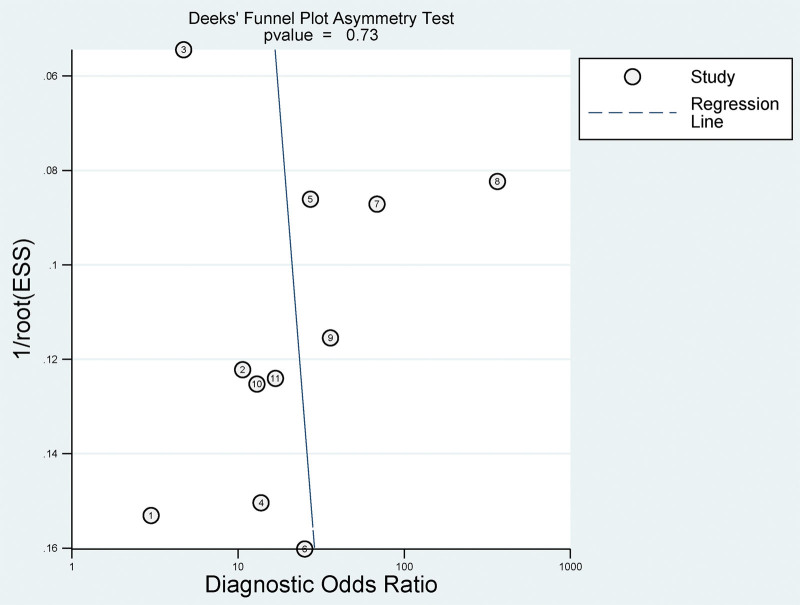
Deek funnel plot for publication bias in all included studies.

Publication bias in the included studies was assessed using Deeks symmetry test. The outcome of the test exhibited a *P* value of .73, which indicates a lack of substantial evidence to confirm asymmetry in the funnel plot, and no significant publication bias was detected in the funnel plots (Fig. 7).

## 4. Discussion

Lung nodules pose significant diagnostic challenges. Current imaging techniques are unable to provide an objective diagnosis; instead, they rely on characteristics such as shape, density, growth patterns, and blood supply to assess the risk of malignancy. This approach has inherent subjectivity and inaccuracies. Small nodules often lack sufficient information to guide treatment decisions, leading to delayed intervention and possibly a delay in diagnosis. The search for a minimally invasive method to accurately diagnose lung nodules remains a clinical challenge. Increasing evidence suggests that miRNAs play a vital role in tumor development and metastasis by suppressing downstream gene expression.^[19,20]^ Studies have detected changes in miRNA levels in the tissues, organs, secretions, and peripheral blood of patients with lung tumors. A ten-year cohort study revealed high expression of serum miRNAs during lung cancer progression.^[21]^ Common sources of miRNAs, such as tissue miRNA, secretory fluid miRNA, and circulating miRNA, can be extracted with minimal harm to the body, making them ideal for early diagnosis.

This study systematically investigated the diagnostic utility of circulating miRNAs for distinguishing between benign and malignant lung nodules. The findings derived from a meta-analysis revealed a promising role for circulating miRNAs in lung nodule diagnosis. With an amalgamated sensitivity of 0.83 (95% CI: 0.72–0.90), the test correctly identified the condition in 83% of affected individuals. The consolidated specificity was 0.81 (95% CI: 0.73–0.88), signifying that healthy individuals were accurately diagnosed 81% of the time. The likelihood ratios further underscore the test’s diagnostic reliability, with a PLR of 4.45 (95% CI: 3.03–6.54) and an NLR of 0.21 (95% CI: 0.12–0.35), emphasizing the robustness of confirming and excluding the condition, respectively. The DOR, an indicator that balances sensitivity and specificity, was 21.31 (95% CI: 10.25–44.30), reflecting the effectiveness of the test in differentiating between disease states. This is further supported by an AUC of 0.89 (95% CI: 0.86–0.91), highlighting commendable overall accuracy. Traditional diagnostic methods, such as LDCT and 18F-FDG PET/CT, have limitations that circulating miRNAs may overcome, particularly in terms of specificity. Circulating miRNAs demonstrate higher specificity in identifying the nature of lung nodules, albeit with a relatively lower sensitivity. Utilizing circulating miRNAs in conjunction with existing imaging methods has been shown to enhance accuracy, and studies have suggested potential applicability in postoperative metastasis detection in head and neck squamous cell carcinoma.^[22,23]^ The publication bias assessment confirmed the absence of substantial evidence of asymmetry, supporting the integrity of the meta-analysis findings. Compared to conventional imaging methods, the measurement of circulating miRNA expression causes less harm to patients.

Apart from miRNAs, other common tumor markers, such as carcinoembryonic antigen, neuron-specific enolase, and CYFRA21-1, are used in diagnosis. miRNA expression is closely related to tumor size and density. He et al^[13]^ showed higher miRNA diagnostic positive rates for nodules > 8 mm in size. The study also revealed varying diagnostic sensitivities among different pathological types of lung cancer, with invasive adenocarcinoma showing the highest sensitivity. In a study by Wang et al,^[18]^ the sensitivity of circulating miRNA diagnosis for nodules > 2 cm in diameter improved from 37.2% to 63.6%, showing a higher diagnostic accuracy for various sizes and densities of tumors than CT. Through subgroup analysis, we found that serum-sourced miRNAs showed higher diagnostic efficiency than plasma-sourced miRNAs. Circulating miRNAs were also found to have a stronger ability to diagnose benign and malignant lung nodules in non-Asian populations, possibly because miRNA screening is more applicable to non-Asian populations.

This study has some limitations, including the exclusion of African populations, which potentially limits the generalizability of the results. Inconsistent diagnostic thresholds across studies and a lack of reporting in approximately 50% of the documents create challenges in comparability and transparency. Additionally, most documents did not report methods to establish diagnostic criteria, thus affecting the evaluation validity. These issues collectively highlight the need for standardized criteria, broader population inclusion, and transparent reporting in future research to strengthen the application of circulating miRNAs in the diagnosis of lung nodules.

## 5. Conclusion

In summary, circulating miRNAs have diagnostic value in distinguishing between benign and malignant lung nodules. Compared to traditional imaging techniques, the use of circulating miRNAs offers higher specificity and less harm to the patient. When combined with existing imaging methods, diagnostic accuracy is further enhanced.

## Author contributions

**Data curation:** Li Liu, Xiaozhao Zou, Zhong Wang.

**Formal analysis:** Yan Nan.

**Investigation:** Li Liu.

**Methodology:** Fei Wang.

**Software:** Dan Jiang.

**Supervision:** Zhong Wang.

**Writing – original draft:** Li Liu.

**Writing – review & editing:** Zhong Wang.
